# Deep Learning Super‐Resolution Spectrometer Based on Fiber Random Laser With Ultrahigh Spectral Purity

**DOI:** 10.1002/nap2.70007

**Published:** 2026-01-14

**Authors:** Jinjiang Zhao, Xiaomei Gao, Zilong Lu, Feng Zhang, Xiaoyu Shi, Tianrui Zhai

**Affiliations:** ^1^ School of Physics and Optoelectronic Engineering Beijing University of Technology Beijing China

## Abstract

The super‐resolution technique based on random laser (RL) achieve the spectra that break through the frequency resolution limit of the original spectrometer. However, the speed of super‐resolution spectrometer methods based on RL is limited by the time‐consuming need to record many thousands of sub‐resolution sparse spectral frames. Here, we propose a deep learning super‐resolution spectrometer based on fiber random laser with ultrahigh spectral purity, obtaining super‐resolution images from up to an 80% reduction in reconstruction time compared with what is usually needed. By coupling to a nested fiber microcavity, the decay rates of RL quasi‐modes broaden, resulting in an excellent micro–nano light source for a super‐resolution spectrometer showing high spectral purity, good directivity, and a miniature size. Based on this micro–nano light source, the sparse frames for reconstructing super‐resolution spectra decreased threefold compared with that reported before. Furthermore, a convolutional neural network is demonstrated to recover the super‐resolution spectra from an 80% smaller number of raw frames or an 80% smaller density of localizations. The drastic reduction in the acquisition time of the super‐resolution spectrometer promotes the development of integrated, low‐cost, high‐resolution spectroscopy with a small footprint.

## Graphical abstract

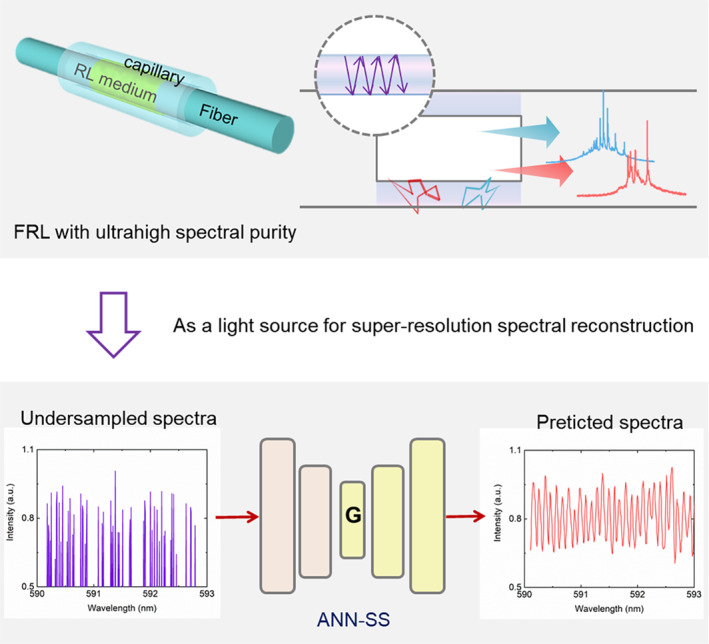

## 1 Introduction

Spectra that convey analyte information are the fundamental and effective characterization tool [1, 2]. Despite multiple attempts to increase a spectrometer’s resolution by optimizing the hardware structure, the Rayleigh criterion limits the accuracy of spectra [3]. Inspired by single‐molecule localization microscopy [4], a super‐resolution spectrometer has been achieved based on coherent random lasers (RLs) [5, 6] as light source by utilizing sparse sampling in the frequency domain [7, 8]. In this method, each spectrum with sharp peaks at random frequencies generates the sub‐resolution spectral response of an analyte stochastically, containing the information only on a few frequencies of the target spectrum. Furthermore, the sharp spikes are uncorrelated in time domain and sparsely distributed throughout the emission bandwidth [9, 10], which enables the accumulation of multiple such spectra to produce reconstructed entire spectra that are finer than the resolution of a spectrometer. Recently, fiber‐based random laser (FRL) in the communication band has been successfully applied to the super‐resolution spectrometer [5]. However, high‐resolution spectrometers are still plagued with the low speed of spectra reconstruction, caused by the long time required for collecting the large number of the sub‐resolution sparse spectral frames.

Two main problems restrict the realization of super‐resolution spectra reconstruction: (i) the traditional random laser suffers from low spectral purity (low contrast of the peaks to the fluorescence background and too dense sharp peaks) [11]. The dense peaks cause overlaps between peaks in the sub‐resolution spectral frames and hinder precise localization of individual peaks. It has been demonstrated that optical cavities could manipulate the mode interactions and the spectra [12, 13]. Therefore, new design should be proposed by introducing micro‐cavities with a high *Q* factor into the RL light source of the spectrometer to promote the further application of the instrument. (ii) In practice, typically 10^3^–10^4^ sub‐resolution spectral frames are needed to assemble one single super‐resolution spectrum [7, 8]. The large number of required sparse spectra makes the super‐resolution spectrometer inherently slow, which limit its potential for high‐throughput spectra with super‐resolution. In recent years, deep learning has been employed to accelerate image acquisition in single‐molecule localization microscopy (SMLM), which reduces the number of single‐molecule image frames required for SMLM reconstruction [14]. The ANNA‐PALM neural network has predicted the super‐resolved image from a small set of frames with incomplete structural features [15]. Deep‐STORM has predicted single‐emitter positions from high‐density data to obtain super‐resolution images from shorter SMLM movies [16, 17]. Deep learning tools are expected to accelerate the super‐resolution spectrometer.

Here, we propose a deep learning super‐resolution spectrometer based on fiber random laser with ultrahigh spectral purity. Firstly, a nested fiber structure is designed to provide high *Q* feedback for random laser through mode coupling. In response to micro‐cavity, the decay rate distribution of quasi‐mode broadens, resulting in the mode separation from the inner coupled ones, allowing the RL spectra to be shaped to fewer well‐separated and uncorrelated peaks with ultra‐high optical signal‐to‐noise ratio. Based on the excellent light source, the sparse frames for reconstructing super‐resolution spectra decreased to 1000, which is 2–3 times smaller than that reported before. Secondly, we first explore the possibility of using a neural network to decrease the sparse frames. By applying a convolutional neural network (ANN‐SS) to learn complex nonlinear mappings from numerical inputs and outputs, the total number of sparse frames is reduced to 20% of the original amount. This work massively accelerates super‐resolution spectral reconstruction, promoting the development of integrated, low cost, high‐resolution spectroscopy with small footprint.

### 1.1 RL Light Source With Ultra‐High Spectral Purity

**FIGURE 1:**
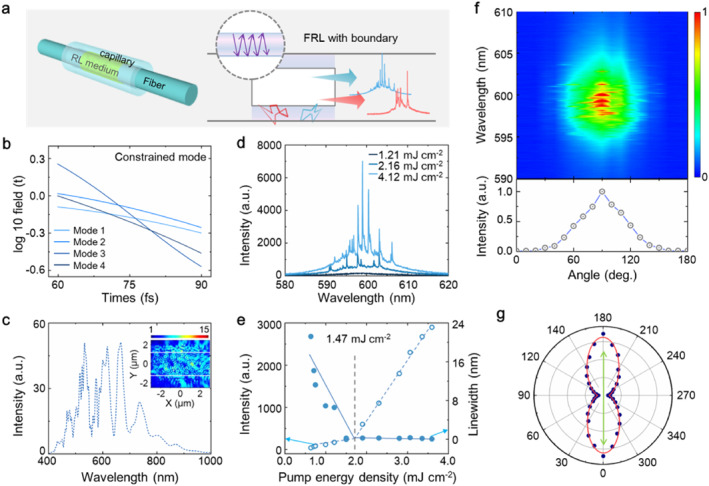
(a) The schematic diagram of the RL coupled with nested fiber cavity. (b) The decay rates of quasi‐modes from the scattering medium coupled with nested fiber cavity. (c) The calculated scattering spectrum of the scattering medium coupled with nested fiber cavity. (d) The emission of the random laser coupled with nested fiber cavity obtained under different pump power densities. (e) Emission intensity and linewidth of the random lasing mode in a nested fiber structure at 601.54 nm versus the pump energy density. (f) The evolution of spectra (up) and integrated intensity (bottom) of the nested FRL as a function of the detection angle. (g) The polarization property of the nested FRL. The green arrow represents the polarization direction of pump light.

In order to obtain coherent RL with high spectral purity, the key idea is to break mode coupling correlation through controlling decay rate of the quasi‐modes [18, 19]. The optical fibers with the waveguide structure can not only confine and guide the emission for directional emission but also act as a micro‐cavity to provide additional feedback for random laser [11, 20, 21]. We design a nested microcavity in the FRL system as shown in Supporting Information S1: Figure S1 and Figure 1a. The hollow fiber is coaxially put on the outside of the optical fiber. In order to realize dynamic spectra with uncorrelated random modes in time domain, liquid matrix with fluidity is necessary, which could lead to large fluctuated emission. Rhodamine B (RhB) as gain media and titanium dioxide (TiO_2_) as the scatterers comprise of the disordered gain medium. To reduce the influence of solvent evaporation on the experiment, glycerinum that hardly evaporates is chosen as the matrix. RhB and TiO_2_ in glycerinum is filled in the air interlayer of the optical fiber and hollow‐core fiber. The outer wall of the optical fiber and the inner wall of the hollow‐core fiber jointly provide additional feedback of FRL system and enhance the *Q* factor.

The effect of nested microcavity on quasi‐modes in FRL based on the steady‐state mode theory is studied [22, 23] in a two dimensional (2D) model. To simulate the quasi‐modes of RL in the nested fiber cavity, the model is built up by a RL media sandwiched between the two uniform media (see in the Supporting Information S1 and Figure S2). The transverse magnetic (TM) quasi‐modes of the model are simulated by exploiting the finite‐difference time‐domain (FDTD) method. Due to the feedback provided by the nested cavity, the decay rate of the scattering medium presents a wide distribution, as illustrated in Figure 1b. The diverse decay rates make quasi‐modes gradually separate from the coupled collective, decreasing the energy loss of mode coupling and enhancing the electric field of quasi‐modes (*E*/*E*_0_ = 15, shown inset of Figure 1c). Accordingly, quasi‐modes with narrow linewidth appear on the spectrum with randomly spaced frequency between adjacent modes (Figure 1c). For comparison, the quasi‐modes in traditional FRL in the Supporting Information S1 indicates that the decay rates of quasi‐modes are basically the same. Consequently, the nested fiber cavity breaks down the mode interaction of RL, which enables the spectra of FRL to be shaped into a perfect state that exhibits very few well‐separated and uncorrelated peaks with a high signal to noise ratio.

The effect of nested fiber cavity on shaping spectra of RL is verified in experiments. As the corresponding emission spectrum is demonstrated in Figure 1d, there is only a spontaneous emission when the pump energy density is 1.21 mJ cm^−2^. By increasing the pump power density to 2.16 mJ cm^−2^, a few sharp peaks with linewidth of 0.2 nm emerge on the emission spectrum. This indicates that the quasi‐modes with small energy loss are selected and enlarged by gain, which is assisted by the nested micro‐cavity feedback. As the pump power density surpasses 4.12 mJ cm^−2^, the intensity increases continuously, whereas the linewidth remains unchanged. The threshold behavior of the nested FRL indicates that the threshold is 1.47 mJ cm^−2^ (Figure 1e). Furthermore, the spatial emission performance of the nested FRL is investigated. By changing the detection angle from 0° to 180° under a fixed pump condition, the emission spectra reach the maximum value at the center (Figure 1f and Supporting Information S1: Figure S3). In addition, the integrated intensities of the spectra increase from the sides to the center. The small emission angle demonstrates that this laser not only has good directivity, but also maximizes collect the energy of random laser and reduces the energy loss. Finally, the polarization property of the nested FRL is plotted in Figure 1g, showing strong dependence on pump light. To highlight the important role of the nested micro cavity for ultrahigh spectral purity of RL, we next aim to conduct further studies (see the Supporting Information S1: Figures S4–S6). To this end, we confirm the important role of nested micro‐cavity for shaping spectra of RL by manipulating quasi‐modes coupling state, outputting directional laser with high purity spectrum.

**FIGURE 2:**
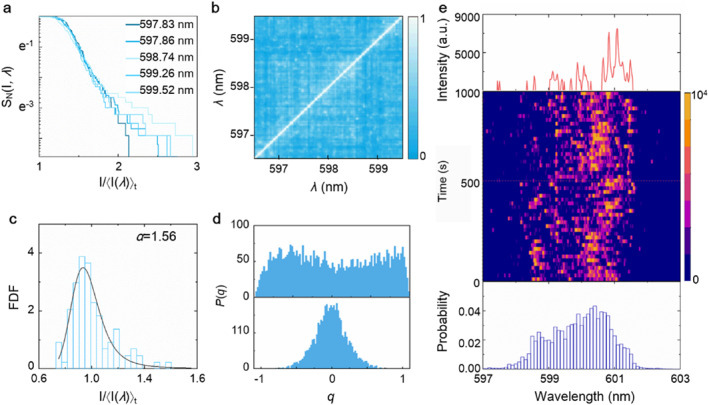
(a) The lasing mode survival functions of the FRL in a nested structure. (b) The Pearson coefficient evaluated from the spectral intensities of FRL in a nested structure. (c) The distribution of emission intensity of FRL in a nested structure (at 588.24 nm). The solid lines are the fit to the *α*‐stable function. (d) Distribution of the Parisi overlap parameter of the FRL at pump energy density of 0.3 mJ cm^−2^ (bottom) and 4.3 mJ cm^−2^ (top) in a nested structure hollow‐core fiber. (e) Evolution of the random wavelength by collecting *N* = 1000 lasing spectra. The spectrum in the top row is one of the collected spectra by removing the fluorescent background. The picture in middle row is the 100 spectra from the 1000 collected spectra. The bottom row is the statistical distribution of the emitted laser modes from the 1000 collected spectra. All the data except (d) are collected at the pump energy density of 4.3 mJ cm^−2^.

The important effect of the boundary on manipulating FRL modes is further studied through statistical method. We record sets of *N* = 1000 single‐shot spectra while all the experimental conditions are kept constant for the replica condition. Firstly, the mode correlation is quantified using the survival function S_*N*_ (*I*, *λ*), which is based on the intensity fluctuations of the emission as plotted in Figure 2a. The survival function S_*N*_ (*I*, *λ*) is defined as the probability (*P*) of emission intensity *I*(*λ*) higher than intensity *I*_0_.

(1) $$S_{N}\;\left(I,\lambda \right)=P\left(I>I_{0}\right),$$

where *I*(*λ*) is the emission intensity at the wavelength of *λ*, *I*_0_ is the shot‐averaged intensity [24, 25]. The RL modes manipulated by nested fiber cavity shows that S_*N*_ for different modes tends to behavior different gradually, indicates the RL modes independently oscillate without interaction. To further characterize the inner correlation of the FRL modes, we quantify explicit correlations between different modes using the Pearson coefficient (*P*) [26, 27], as shown in Figure 2b. The Pearson correlation is around 0.19, indicating independently oscillating state of modes. Next, the fluctuation of emission intensity on a time scale is statistical analyzed by fitting to *α*‐stable distributions [28]. The *α*‐stable distribution is conveniently described by the characteristic function *φ*(*t*),

(2) $$\ln\;\phi \left(t\right)=-\sigma^{\alpha }{\left|t\right|}^{\alpha }\left\{1-i\beta \text{sgn}\left(t\right)\tan\frac{\pi \alpha }{2}\right\}+i\mu t\;\text{for}\;\alpha \neq 1,$$

where *μ* is the location parameter, *β* is the skewness parameter, and *σ* is the width. The output intensity fluctuates strongly (in Supporting Information S1: Figure S7a), which demonstrates a typical heavy tailed distribution. The fits of *α*‐stable distributions yielded the tail exponents *α* = 1.56, indicating Levy behavior (in Figure 2c and Supporting Information S1: Figure S7b). To further qualify the spectral fluctuation of FRL in a period of time, the overlap parameter ($q_{\gamma \beta }$) is exploited [29]. The *P*(*q*) distribution for the emission above threshold is broadened, with shoulders appearing in the distribution near *q*_max_ ∼ 0.6, suggesting that the modes are fluctuating in real‐time and uncorrelated (in Figure 2d and Supporting Information S1: Figure S7c). The results are fundamentally consistent with the theoretical prediction. These two features (sparsity) are important and necessary for the super‐resolution spectrometer.

Despite the uncorrelated and time‐to‐time random fluctuated performance, the wavelength range covered by the RL modes (ergodicity) is another important factor for super‐resolution spectrometer. The overall wavelength distribution of peaks in nested FRL spectra is studied. We collected *N* = 1000 lasing spectra at the pump energy density of 4.3 mJ cm^−2^, as shown in Figure 2e and Supporting Information S1: Figure S8. The evolution of spectra (in the middle of Figure 2e) indicates that the wavelength positions of peaks in each spectrum are randomly changing and cover the whole emission band together. The upper picture of Figure 2e is one of the above spectra, which is recorded at 500 s. Furthermore, the statistical distribution of the emitted laser modes is evaluated by accumulating all the probability of laser peaks, as plotted in Figure 2e. Obviously, the average number of peaks over the emission band validates that the nested FRL satisfies the second essential requirements (ergodicity) for the super‐resolution spectrometer.

### 1.2 Super‐Resolution Spectra Reconstruction

**FIGURE 3:**
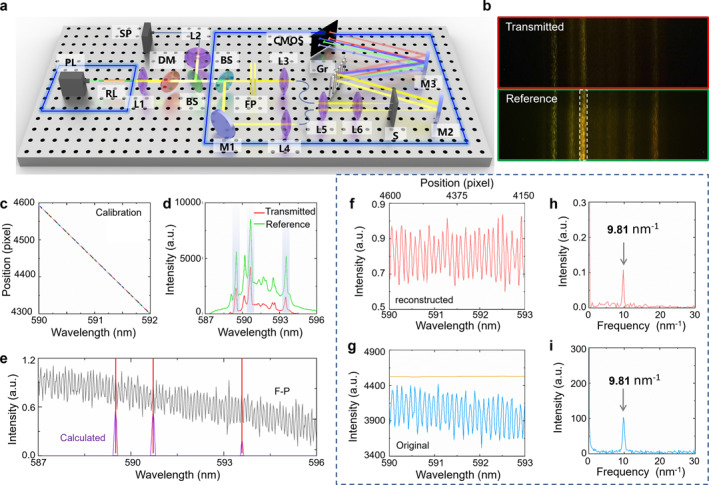
(a) Schematic of the experimental setup. BS, beam splitter (50:50); CMOS, complementary metal‐oxide semiconductor camera; DM, dichroic mirror; FP, Fabry−Perot filter; Gr, grating; L, lens; M, mirrors; PL, pump laser; SP, spectrometer. (b) The low‐resolution spectra measured by the CMOS camera under illumination of random laser. The top and bottom rows correspond to the transmitted (red) and reference (green) signal, respectively. (c) The relationship of input wavelengths and their center positions of light spots on the CMOS camera. (d) The corresponding spectra of (b). (e) The sparse spectrum of the FP sample calculated by the ratio of the two spectra in (d). (f) Reconstructed transmission spectrum of the FP sample by sparse sampling illuminated by the random laser. (h) FFT of the spectrum in (f), showing a prominent peak at 9.81 nm^−1^. (g) Transmission spectrum of the FP sample recorded by a high‐resolution (blue curve) and low‐resolution spectrometers (yellow curve), which are illuminated by a broadband lamp. (i) FFT of the spectrum (blue curve) in (g), showing a prominent peak at 9.81 nm^−1^.

As a proof‐of‐concept, experimental demonstration of super‐resolved spectroscopy based on this RL with high spectral purity is proposed. The experimental setup is shown in Figure 3a, which includes two parts, which are the RFL for illumination and the setup for spectral measurement. The spectral profile is captured as the spectral image shown in Figure 3b. The spectral image contains the transmitted signal (top) and its reference signal (bottom), representing the RL emission and the transmitted light modulated by sample, respectively. The spectral profile in the image is well separated, enabling us to precisely determine the emission wavelength. It demonstrates that the wavelength‐sparse sampling avoids overlapping interference of adjacent wavelengths. To convert the spectral image to a spectrum, we calibrated using a wavelength scanning laser, as the calibration relationship between the wavelengths and the central positions of light spots shown in Figure 3c. The corresponding spectra of Figure 3b is derived by integrating the light intensity over the longitudinal spot, as illustrated in Figure 3d. The target modes of FRL are selected by setting the limit of the signal‐to‐noise ratio and line width of the isolated peak. Thus three peaks are selected from the spectrum with multiple peaks. Then, we calculate the transmittances of the selected modes from the intensity ratio between the probe and the reference spectrum, as shown in Figure 3e, which is right at the peak of the transmission spectrum of FP sample (gray line). To fully cover the detection range, we repeat this sparse wavelength sampling by collecting a set of 2000 uncorrelated single‐shot spectral images in 2.5 min. By the algorithm evaluating sufficiently the wavelengths and intensities of all valid spectra, the whole transmission spectrum of the FP sample is reconstructed in Figure 3f, showing all the relevant features. We also calculate the power Fourier transform (FFT) of the reconstructed spectrum, showing a dominant peak at 9.81 nm^−1^ (Figure 3h). To verify the accuracy of the reconstructed spectrum, we record the exact transmission spectrum of the FP sample by the high‐resolution spectrometer (resolution of 0.01 nm) in Figure 3g, which demonstrates the spectral features of the FP sample is successfully recovered. Furthermore, we measure the transmission spectrum by the low‐resolution spectrograph illuminated by a standard lamp for comparison. The spectral resolution of the low‐resolution spectrograph is 0.173 nm, which is determined by its instrumental line profile. It is much larger than the sample’s FSR (0.1 nm). The transmission spectrum recorded by low‐resolution spectrograph only reflects the envelope profile of the FP modulated spectrum, which lacks spectral details, as the yellow curve illustrated in Figure 3g, which is because convolution with the instrumental response function (yellow line in Figure 3). The FFT of the spectrum in Figure 3g reveals a prominent frequency peak at 9.81 nm^−1^ (Figure 3i), which is in accordance with the result of the wavelength‐sparse sampling approach. As confirmed by our results, the spectral reconstruction‐based FRL can obtain features of a transmission spectrum below the resolution limit of the spectrometer, with spectral enhancement of ∼6.9.

### 1.3 Deep Learning Super‐Resolution Spectra Spectrometer

**FIGURE 4:**
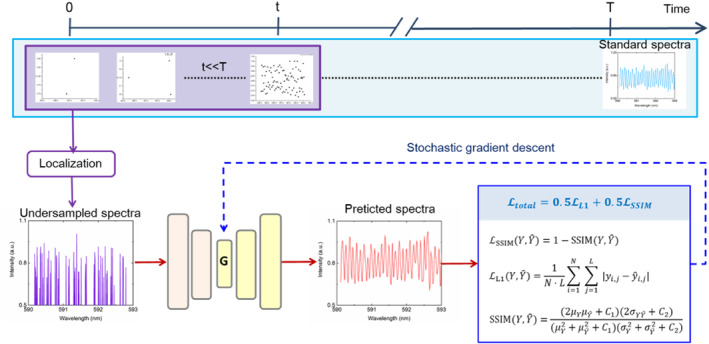
The artificial neural network (ANN) training. Training spectra are reconstructed by traditional algorithms from a few long sequences. The acquisition time for each image sequence is *m*Δ*t*, where Δ*t* is the single‐frame exposure time. For each experiment, a standard spectrum is generated using all the *M* frames. Sparse spectra are obtained by using only *m* << *M* frames. The ANN (labeled G) is trained by using the sparse spectra as inputs and the corresponding standard spectrum as target output. During training, the output of the generator G is compared to the target spectra via loss functions.

**FIGURE 5:**
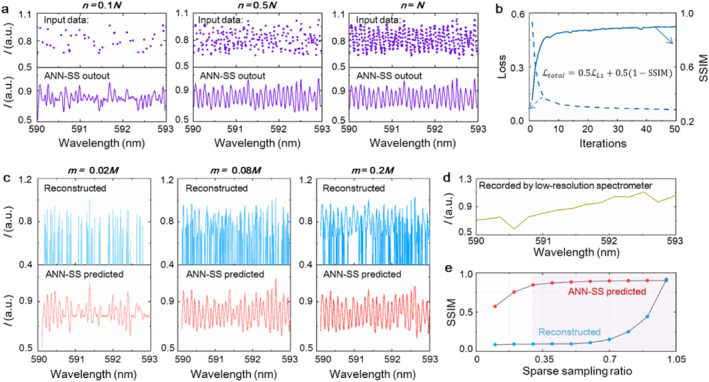
(a) The input locations (top row) for ANN‐SS and their predicated spectra obtained from different locations. (b) The value of loss and SSIM for the testing data. (c) The under sampled spectra reconstructed by traditional method and ANN‐SS predicated spectra obtained from different frames. (d) The spectrum obtained by the spectrometer with resolution of 0.4 nm. (e) Reconstruction quality of reconstructed spectra based on traditional method and ANNA‐SS predicted spectra, measured by the SSIM with the standard spectra, as function of frames *m*.

To further accelerate the super‐resolution spectra spectrometer, we aim to reconstruct a super‐resolution spectra (predicated spectra) that approximately similar a standard (dense) spectra (reconstructed with *M* frames and *N* localizations) from a much smaller number of raw frames (*m* << *M*) without changing the average density of localizations (*ρ*), that is, from a much smaller number of total localizations (*n* = *ρm* << *N* = *ρM*). On the basis of artificial neural network (ANN), we propose a deep learning method for recovering standard super‐resolution spectra (dense spectra) from undersampled spectra data (sparse spectra), called ANN‐SS (in Figure 4). Our proposed ANN‐SS modifies the U‐net [30] to a symmetric encoder–decoder structure, consisting of two downsampling stages in the encoder, two upsampling stages in the decoder, and roughly 500 thousand trainable parameters. Unlike the U‐Net, we omit skip connections to reduce computational complexity without affecting the reconstruct high‐resolution signals. The encoder compresses the input sparse signal into a latent representation through strided convolutions, and the decoder progressively recovers the full‐resolution output through transposed convolutions. This design is inspired by fully convolutional networks [31] but tailored for 1D signal processing. The ANN is trained to recover the standard spectra from undersampled spectra. The standard spectra are super‐resolution spectra that obtained by reconstructing low‐resolution spectra sequences (*M* = 2000; as illustrated in Figure 5). In addition, the undersampled spectra are constructed by using a much fewer number of low‐resolution spectra frames (*m* << *M*). Randomly undersampled spectra are fed as input to the A‐net, whereas the corresponding standard spectra are defined as the targets. We implemented a loss function that defines the difference between the A‐net output and the standard spectra. Instead of the widely used mean squared error (MSE), the combination of the absolute difference (L1 norm) with a multiscale version of the structural similarity index (L1‐SSIM) [32] is proposed as the loss function, $L=L_{L1}+\lambda \cdot L_{\text{SSIM}}$. The L1 norm calculates the absolute pixel‐wise differences between the predicted spectrum $y_{i}$ and the ground truth y, which is defined as follows:

(3) $$L_{L1}=\frac{1}{n}\sum_{i=1}^{n}|{\hat{y}}_{i}-y_{i}|.$$

Compared with mean squared error (MSE), the L1 norm is more robust to outliers and promotes sparser reconstructions, which can accurately recover sharp peaks in the spectra without excessive smoothing or blurring. The 1D structural similarity index (SSIM) loss is adapted to preserve the global structural integrity of the spectrum, which is defined as follows:

(4) $$L_{\text{SSIM}}=1-\text{SSIM}\left(\hat{y},y\right).$$

The SSIM between two 1D signals is computed as follows:

(5) $$\text{SSIM}\left(x,y\right)=\frac{\left(2\mu_{x}\mu_{y}+C_{1}\right)\left(2\sigma_{xy}+C_{2}\right)}{\left(\mu_{x}^{2}+\mu_{y}^{2}+C_{1}\right)\left(\sigma_{x}^{2}+\sigma_{y}^{2}+C_{2}\right)},$$

where μ and σ represent the local mean and standard deviation, $\sigma_{xy}$ is the local covariance, and $C_{1},C_{2}$ are small constants for numerical stability. Thus the SSIM loss ensures that the reconstructed spectrum maintains the correct overall shape, contrast, and relative intensity relationships between spectral peaks. Therefore, the L1 loss excels at preserving local details (sharp peaks), whereas the SSIM loss ensures global structural fidelity (spectral envelope). The loss function effectively balances these objectives, enabling the model to reconstruct spectra that are both locally precise and globally consistent.

The ANN‐SS is trained on simulated data, and being tested on both of our simulated data and experimental data. We apply varying levels of Poisson noise to a constructed high‐resolution spectrum (in Figure 3f) to generate 500 standard spectra. Then the undersampled spectra are created by randomly select part of the data points (*n* are set as 0.1*N*, 0.2*N*, 0.3*N*, 0.4*N*, 0.5*N*, 0.6*N*, 0.7*N*, 0.8*N*, 0.9*N,* and *N*) from the standard high‐resolution spectrum. The ANNA‐SS is trained using the 500 standard spectra as targets and the 500 undersampled spectra as inputs. After being trained, the ANN‐SS is applied to new simulated undersampled spectra as input, and output a reconstructed super‐resolution spectrum in less than a second. The top row in Figure 5a and Supporting Information S1: Figure S9 shows the input locations, and the bottom row are corresponding undersamples spectra obtained from different locations (*n* from 0.1*N* to *N*). The ANNA‐SS spectra reconstructed from the undersampled locations exhibit sharp peaks in good agreement with the standard spectra when the locations are beyond 0.2*N* (Supporting Information S1: Figure S9). The results indicate that high‐quality, super‐resolution spectra can be predicted from only a small number of localizations traditionally required (∼20% of *N* above), hence enabling a strong reduction in acquisition time. Figure 5b shows the value of loss functions and SSIM for the test data, indicating that the ANN‐SS model works well. ANN‐SS achieves the same L1‐SSIM as standard reconstructed spectra at the 80% sampling level, suggesting a 5‐fold increase in speed. We next tested ANNA‐SS on real experimental spectra by inputting part of the sparse frames (with *m* = 0.02*M*, 0.04*M*, 0.08*M*, 0.1*M*, 0.2*M*). Although some peaks can already be seen in this undersampled (sparse) spectra, most details are hard to discern (in the top row of Figure 5c and Supporting Information S1: Figure S10), making it difficult to identify F‐P cavity features. In contrast, the ANNA‐SS predicted spectra display sharp and evident peaks (in the bottom row of Figure 5c and Supporting Information S1: Figure S10). Their resolution is similar to the standard (dense) spectra (in Figure 3f) with only 400 sparse frames (20% of *M*). Figure 5d shows the spectrum obtained from the low resolution spectrometer, which exhibits vague spectral characteristics with many small features being erroneous. To quantify the reconstruction errors, the SSIM between ANNA‐SS predicated spectra and standard spectra (*m* = 2000) as a function of localization number, from *m* = 40 to *m* = 400. For the spectra reconstructed by traditional method, the L1‐SSIM increases monotonically from 40 to 2000 ( blue dots in Figure 5e). The SSIM of ANN‐SS predicted spectra are consistently higher and increase with frame number *m*, exceeding 0.9 for *m* > 0.2*M* (red dots in Figure 5e). Figure 5e indicates that reconstruction quality increases with the number of frames *m*. Based on the L1‐SSIM analysis, it can be demonstrate that ANNA‐SS allows a 80% reduction of acquisition time compared to the standard method (see in Table 1).

**TABLE 1:** Comparison of processing times for different spectrometers.

Super‐resolutions spectrometer	Frame numbers	Resolution enhancement	Time for spectra collecting	Time for spectra reconstruction
Reference [7]	4000	2.8	5 min	—
Reference [8]	3000	3.3	A few minutes	—
Based on nested FRL	2000	6.9	2.5 min	
Deep leaning and nested FRL	400	6.9	20 s	5 s

*Note:* Green, conventional super‐resolution spectrometer; Red, proposed deep learning‐assisted super‐resolution spectrometer.

## 2 Conclusion

In conclusion, super‐resolution spectra reconstruction is massively accelerated by RL light source with high spectral purity and a deep learning method. By utilizing a nested‐fiber micro‐cavity to decouple the RL quasi‐modes, the RL spectra are shaped to very few well‐separated peaks with narrow linewidth and high signal‐to‐noise ratio. Based on this FRL as the light source, the number of sparse spectra frames for super‐resolution spectra reconstruction is decreased to 2000, resulting the time being reduced by half. Further, a deep leaning tool is first introduced to accelerate super‐resolution spectra prediction. The ANN‐SS model is proven to recover the super‐resolution spectra that are approximately similar to the standard spectra from an 80% smaller number of raw frames or an 80% smaller density of localizations, resulting in an 80% reduction in reconstruction time. By using far fewer frames, ANNA‐SS could dramatically improve the temporal resolution of super‐resolution spectrometer without sacrificing frequency resolution. This will facilitate super‐resolution studies of spectra characterization. Thus, ANNA‐SS provides novel avenues for spectroscopy beyond conventional spatiotemporal resolution limits. We envision this to develop into a powerful tool for integrated, low cost, and high‐resolution spectroscopy.

## 3 Methods

### 3.1 Experimental Design

The first part is the RFL for illumination. The pump beam (*Q*‐switched second‐harmonic Nd:YAG pulsed laser, MINILITE II, 532 nm, 10 Hz, 5–7  ns) with a diameter of 1 mm first enters the nested FRL from the end, which is guided along the optical fiber and exits the gain media surrounding the optical fiber. The emission of FRL is collimated through the lens (L1). The emission the passes a dichroic mirror (DM) filter out excess pump light. A 50:50 beam splitter (BS) sends apportion of signal to the second part, whereas the other portion signal is focused to the optical fiber spectrometer (Ocean Optics HR4000) with a spectral resolution of 0.01 nm for spectral analysis. The second part of the setup is the spectrograph for spectral measurement. The FRL emission entering the second part is divided into a probe beam and a reference beam by a 50:50 BS. The reference beam is directly coupled to a multimodal fiber (NA = 0.22), which is assigned to compensate for the fluctuations of the FRL. The probe beam passes through the high‐fitness FP sample (FSR = 0.1 nm) and enters other multimodal fibers. The core diameter of the fiber is 600 μm. The two fibers are bundled together and simultaneously focused at the entrance of a homemade low resolution monochromator (*λ*‐300), which transversely disperses light with different wavelengths, forming the far‐field pattern of the spatial intensity distribution. The spectral profile is captured by a complementary metal‐oxide semiconductor camera (CMOS, Light Conversion TOPAS, 5496 × 3672 pixels).

## Author Contributions

X. Shi and J. Zhao designed the study. X. Shi and T. Zhai performed the data analysis and wrote the paper. J. Zhao assisted in the processing and analysis of data. Z. Lu, J. Zhao and F. Zhang assisted in the processing data. X. Shi and X. Gao carried out the simulation. All authors commented on the paper.

## Conflicts of Interest

The authors declare no conflicts of interest.

## Supporting information


Supporting Information S1

## Data Availability

The data that support the findings of this study are available from the corresponding author upon reasonable request.
