# Supplementary material for: Deep Learning Super‐Resolution Spectrometer Based on Fiber Random Laser With Ultrahigh Spectral Purity
Source: Nanophotonics. 2026 Jan 14;15(1):e70007. doi: 10.1002/nap2.70007 (PMC12965029; doi:10.1002/nap2.70007)
Supplement: Supplementary file 1 — Supporting Information S1 [file NAP2-15-e70007-s001.docx]

**Deep learning super-resolution spectrometer based on fiber random laser with ultrahigh spectral purity**

Jinjiang Zhao, Xiaomei Gao, Zilong Lu, Feng Zhang, Xiaoyu Shi^*^ and Tianrui Zhai^*^

*School of Physics and Optoelectronic Engineering, Beijing University of Technology, Beijing 100124, China.*

**xyshi@bjut.edu.cn*

**trzhai@bjut.edu.cn*

**A: Schematic illustration of the RL coupled with nested fiber cavity**

The random laser (RL) RL coupled with nested fiber cavity is schematically illustrated in Figure S1, which is realized by a nested cylindrical structure. The optical fiber (*n* = 1.45) with the diameter of 660 μm is the core of the nested cylindrical structure. And the hollow-core fiber (*n* = 1.45) with inner diameter of 680 μm acts as the shell of the nested cylindrical structure. The refractive index of cladding and core of the inner optical fiber are 1.445 and 1.457, and the diameter of cladding and core of the inner optical fiber are 660 um and 600 um respectively. RL medium is composed of Rhodamine B (Rh B) and titanium dioxide (TiO_2_) mixed in glycerol (*n* = 1.47) with the concentration of 0.5 mg mL^-1^ and 4.7 ☓ 10^17^ mL^-1^, filling the gap between inner optical fiber and outer hollow-core fiber. The RL medium in the nested structure formed a liquid hollow cylinder with pipe thickness at about 20 μm (as shown in Figure S1). The scattering free path (*l*_s_) of the random medium is estimated as 800 μm, which is 10^3^ larger than the wavelength (~ 600 nm), indicating that the scattering system is diffusive regime.


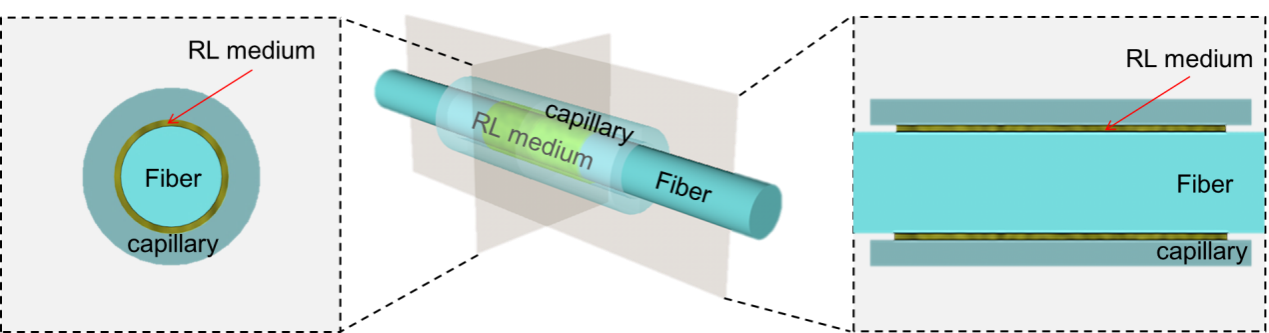


Figure S1 Schematic illustration of the RL coupled with nested fiber cavity. Left: the cross section of the device. Middle: space diagram of the device. Right: longitudinal section of the device.

**B: Simulation of fiber based random laser**

Numerically, the important effect of boundary on controlling quasi-modes of nested fiber based random laser (FRL) is investigated. We conduct our theoretical simulation based on the finite difference time domain method (FDTD). The nested FRL is simplified by a two-dimensional (2D) model, as shown in Figure S2a. Our 2D FRL system is made of random scattering media sandwiched between two rectangular regions. The 2D random scattering media is randomly distributed circles with refractive index of 2.6 and diameters of 100 nm. The middle rectangular medium with a total size of 15×3 μm^2^ is characterized with the refractive index at 1.47. The medium outside the middle rectangular medium is 1. The boundary provide additional feedback for the random scattering, resulting to diverse decay behavior of the quasi-modes, as shown in Figure 1b. The simulated electric-field distribution of the laser mode constrained by nested fiber cavity shows the extended electric-field distribution higher intensity. The quasi-modes in the traditional FRL without additional cavity feedback is subsequently studied by setting the refractive index of the medium outside the middle rectangular medium as 1.47, as is shown in Figure S2b. The decay behavior of the quasi modes is shown in Figure S2c, indicating that the decay rates of quasi- modes are basically the same. As the electric-field distribution of the quasi-modes shown inset of Figure 1d, the modes with smaller intensity (*E*/*E*_0_ = 7) spread to the whole system. The lasing modes are spatially extended and overlapped with each other. Therefore, lasing in most quasi-modes is suppressed for their close frequencies, resulting to depleting the gain in one subgroup of quasi-modes, as the calculated spectrum shown in Figure S2d. The lasing spectrum of the modes shows a main peak with higher intensity. In this case, the emission spectrum of RL is single broad peak or multi-peak with small low contrast to fluorescent background.

**
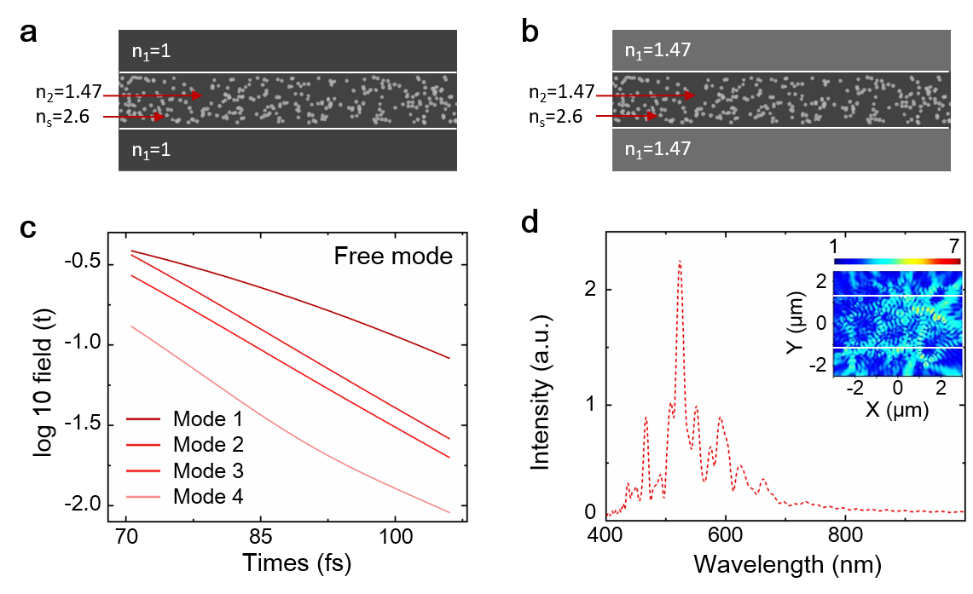
**

Figure S2 (a) Simulation structure for nested-fiber based RL. (b) Simulation structure for traditional FRL. (c) The decay rates of quasi- modes from the traditional FRL. (d) The calculated scattering spectrum of the traditional FRL.

**C: Spatial emission property of nested FRL**

To study the spatial emission characteristics of the nested FRL, the emission close to the end face of the fiber are recorded, as illustrated in Figure S3. By changing the detected angle from 0 to 180°, all the angle resolved spectra (shown in Figure S3) exhibit multiple peaks, showing intensity increase from the sides to the center. It demonstrates that the nested FRL has good directionality.


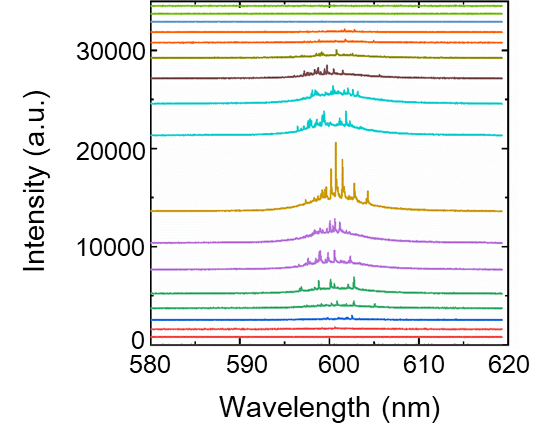


Figure S3 Emission spectra of nested FRL detected from different angles. The pump power density is 4.3 mJ cm^-2^.

**D: Excluding the possibility of whispering gallery modes**

To explore whether the spectra are shaped by whispering gallery mode (WGM) cavity formed by the fibers, we performed a contrast experiment on the nested FRL systems by changing the pump condition. When the nested FRL is side-pumped, there is only weak fluorescence emitted (shown in Figure S4a). And there is no coherent mode emerged in the FRL without the internal optical fibers (in Figure S4b), which further rules out the possibility that (WGM) cavity on the spectral shaping is significant.


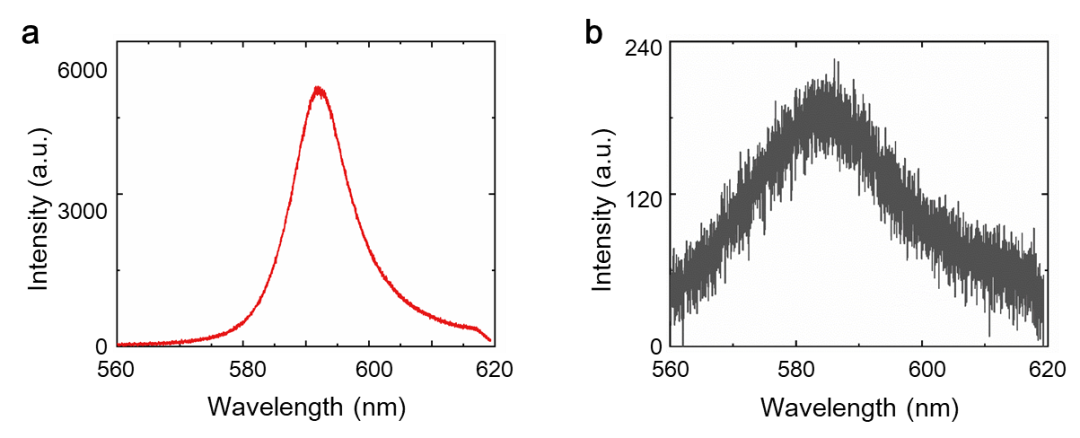


Figure S4 (a) Emission spectrum of the nested FRL obtained by side pump. (b) Emission spectrum of the FRL without the internal optical fibers. The pump power density is 4.3 mJ cm^-2^.

**E: Confirming the effect of boundary for spectral shaping**

To prove whether the nested fiber micro-cavity is attributable to the spectral shaping. We weakens the effects of micro-cavity by decreasing the diameter of the internal optical fibers in nested FRLs, there is no sharp peaks in the emission spectrum (in Figure S5a). This is because the increasing volume of RL media reduces the interaction between micro-cavity and random feedback. The size of the outside hollow fiber only affects the quantity of the gain medium. The gain of the random laser decreases as the diameter of the fiber decreases. The gain medium can not provide sufficient gain to achieve laser operation when the diameter of the fiber is too small. In addition, we alter the nested structure by replacing the surface of the internal optical fibers with roughness structure. The broken surface cannot provide feedback for random laser, the output spectrum in Figure S5b is a broad band peak. The results confirm that the nested micro-cavity is responsible for the spectral shaping of nested FRL.


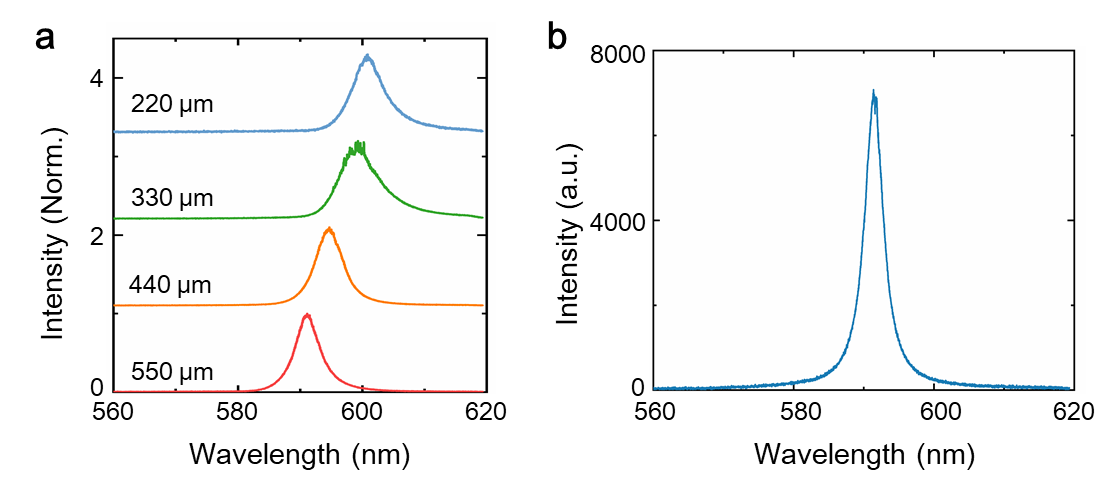


Figure S5 (a) The spectra from nested FRL by decreasing the diameter of the internal optical fibers in nested FRLs. (b) The spectrum from cested FRL with roughness boundary. All the data are obtained from spectra at a pump energy density of 4.3 mJ cm^-2^.

**F: Emission property of nested FRL with different gain and scattering parameters**

We sought to prove that the essence of these discrete modes are random lasers. Emission property of nested FRL with different gain and scattering parameters is studied here. When the concentration of scatterers is changed from 2.9 ☓ 10^16^ mL^-1^ to 1.1 ☓ 10^19^ mL^-1^ while keeping the concentration of RhB fixed at 0.5 mg mL^-1^, all the spectra exhibit discrete narrow linewidth peaks (Figure S6a). There is no coherent RL observed when there is no scatterer in the system. This demonstrates the discrete modes are generated by random feedback. Furthermore, by changing the concentration of RhB from 0.1 mg mL^-1^ to 3 mg mL^-1^, more sharp peaks are emerged. The increased gain leads to more modes reaching threshold to realize lasing (Figure S6b).

**
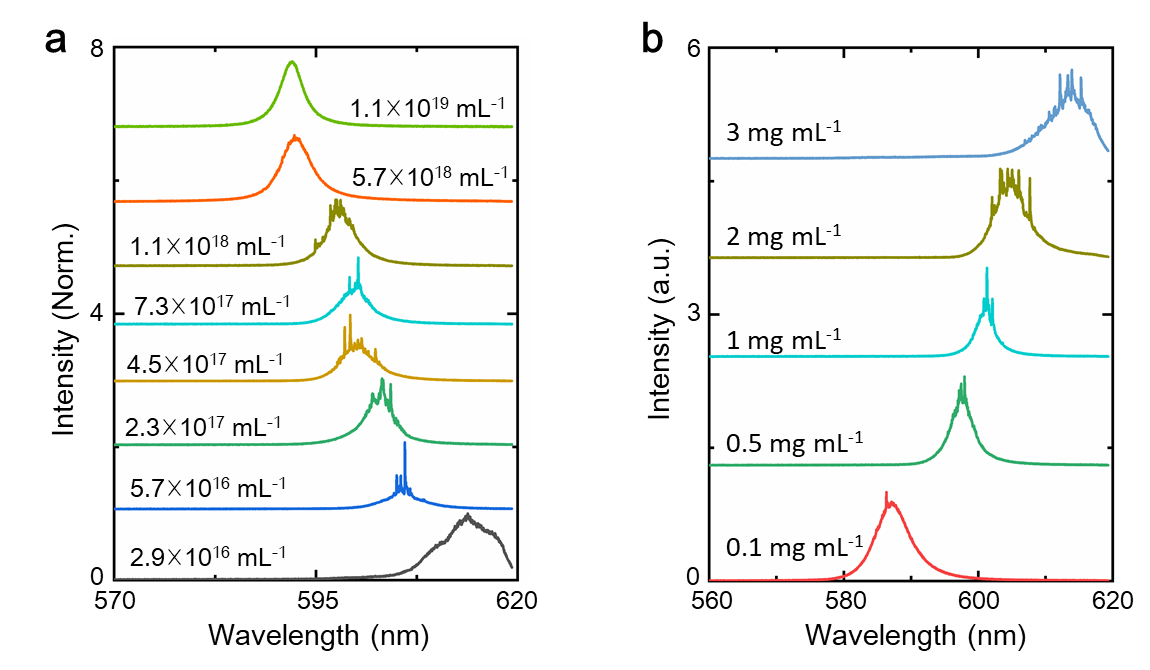
**

Figure S6 (a) The spectra from boundary assisted FRL via different scatterer concentration. (b) The spectra from boundary assisted FRL via different gain concentration. All the data are obtained from spectra at a pump energy density of 4.3 mJ cm^-2^.

**G: Statistical study of nested FRL**

To reveal the dynamic characteristics of the nested FRL, their emission intensity are statistically studied by recording 1000 spectra with fixed experiment condition. Notably, the emission intensity of nested FRL are changeable (Figures S7a). We quantitatively characterize the intensity fluctuation by the ratio of the maximum value to the minimum value of emission intensity, as *β* = *I*_max_/*I*_min_. The intensity fluctuation ratio of the mode at 591.8 nm manipulated by boundary is 15. Furthermore, the corresponding mode correlations of random laser are demonstrated by recording the intensity of two modes at different times. Figure 7b present the correlations of the nested FRL. The random laser modes are almost uncorrelated, showing the randomly distributed blue dots. Furthermore, the corresponding correlations between different replicas are also illustrated in Figure S7c. There replicas are the recorded spectra. By choosing the first spectrum and the last spectrum under fixed pump energy densities, the points with random distribution demonstrate that the two replicas are almost uncorrelated.

The Pearson coefficient (*P*) is calculated by

$$P=\frac{\sum_{i=1}^{N} [{I\left( \lambda_{1} \right)}_{i}-\overline{I}\left( \lambda_{1} \right)][{I\left( \lambda_{2} \right)}_{i}-\overline{I}\left( \lambda_{2} \right)]}{\sqrt{\sum_{i=1}^{N} {[{I\left( \lambda_{1} \right)}_{i}-\overline{I}\left( \lambda_{1} \right)]}^{2}}\sqrt{\sum_{i=1}^{N} {[{I\left( \lambda_{2} \right)}_{i}-\overline{I}\left( \lambda_{2} \right)]}^{2}}}$$

where *I*(*λ*_1_) and *I*(*λ*_2_) represent the intensity values of the modes at *λ*_1_ and *λ*_2_ from *N* = 1000 shots. By trace the intensities of any two lasing modes at the pump energy density of 4.3 mJ cm^-2^ as the time changes, the Pearson correlation from these two peaks is obtained. The value of *P* ranges from 0 to 1, where 1 indicates perfect correlation and 0 indicates no correlation.


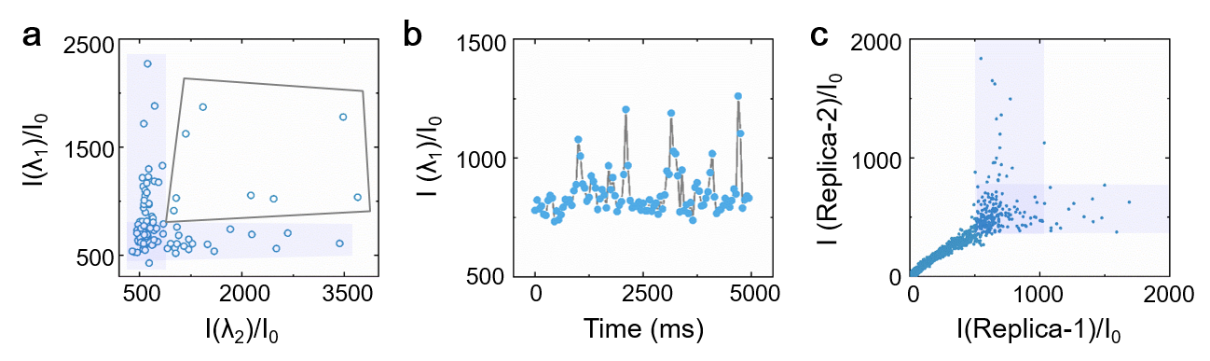


Figure S7 (a) Intensity variation of the lasing modes at 591.8 nm. (b) Phase diagrams of relative intensity values of the random lasing modes at 589.74 and 597.86 nm. (c) Relationship between the emission intensity from the first replica and the last replica of nested FRL. All the data are obtained from spectra at a pump energy density of 4.3 mJ cm^-2^.

**H: Temporal evolution of boundary assisted FRL emission**

The dynamic characteristics of emission spectra in boundary assisted FRL are studied. Under a pump energy density of 4.3 mJ cm^-2^, the spectra with well separated and narrow linewidth peaks are changeable from time to time (Figure S8). This demonstrates the random laser modes from different feedback channels are selected by the boundary. The peaks distribute over the whole emission band. The time-to-time fluctuated spectra with broad emission range meet the requirement of super-resolution spectral reconstruction.

The stability of the emission dose not affect the sparse sampling. For super-resolution reconstruction tasks, the absolute intensity in the spectra with sparse sampling is meaningless. The final reconstruction process only requires the peak positions and relative intensities, which is not related to stability.


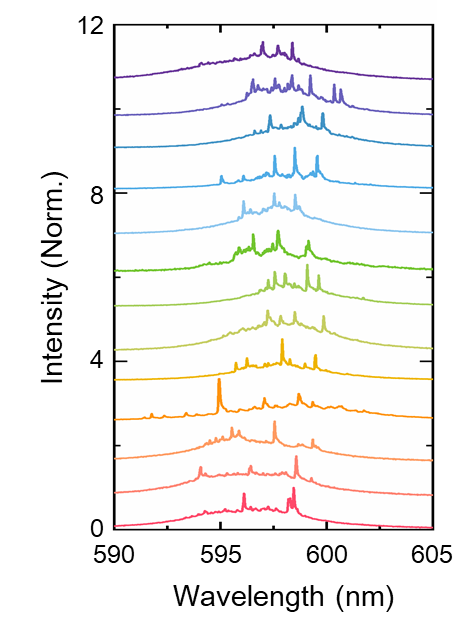


Figure S8 Temporal evolution of boundary assisted FRL emission obtained at a pump energy density of 4.3 mJ cm^-2^.

**I: ANN-SS output with simulates data**

The simulated undersampled spectra as input output reconstructed super-resolution spectra in less than a second. The top row in Figure S9 shows the input locations, and the bottom row are corresponding undersamples spectra obtained from different locations (*n* from 0.1*N* to *N*). The ANNA-SS spectra reconstructed from the undersampled locations exhibits sharp peaks in good agreement with the standard spectra when the locations beyond 0.2*N* (Figure S9).


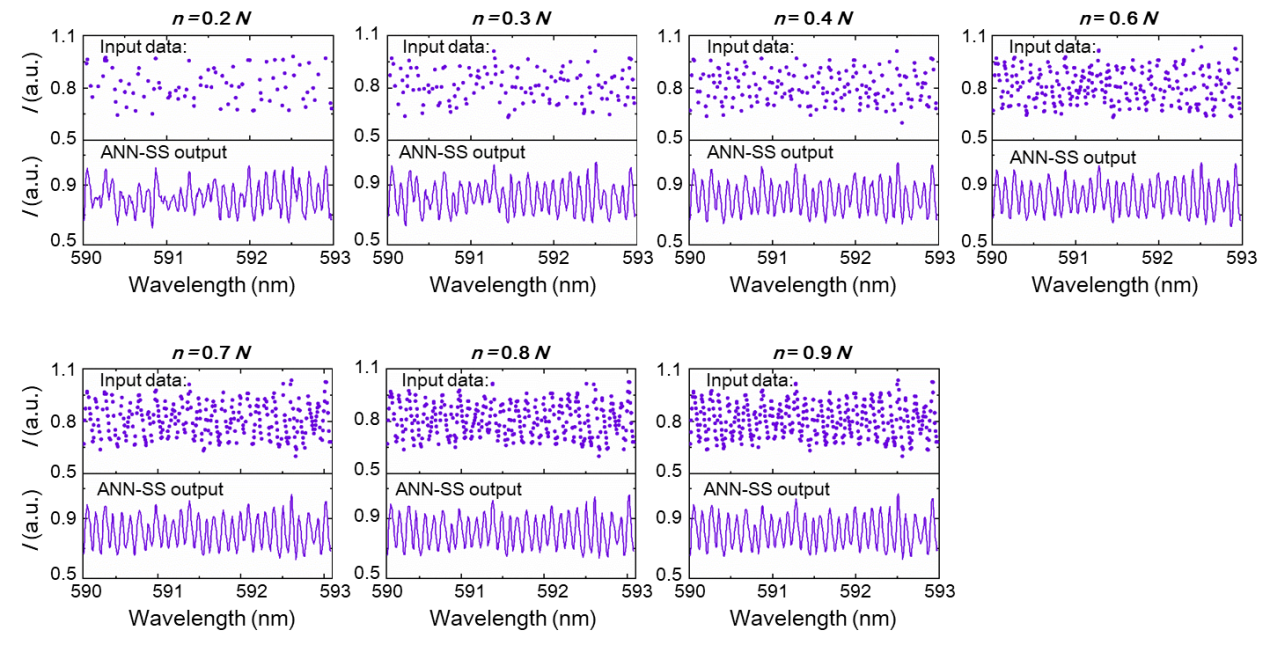


Figure S9 The input locations (top row) for ANN-SS and their predicated spectra obtained from different locations.

**J: ANN-SS output with expimental data**

We next tested ANNA-SS on real experimental spectra by inputting part of the sparse frames (with *m* = 0.04*M*, 0.1*M*). Whereas some peaks can already be seen in this undersampled (sparse) spectra, most details are hard to discern (in the top row of Figure S10), making it difficult to identify F-P cavity features. By contrast, the ANNA-SS predicted spectra display sharp and evident peaks (in the bottom row of Figure S10).


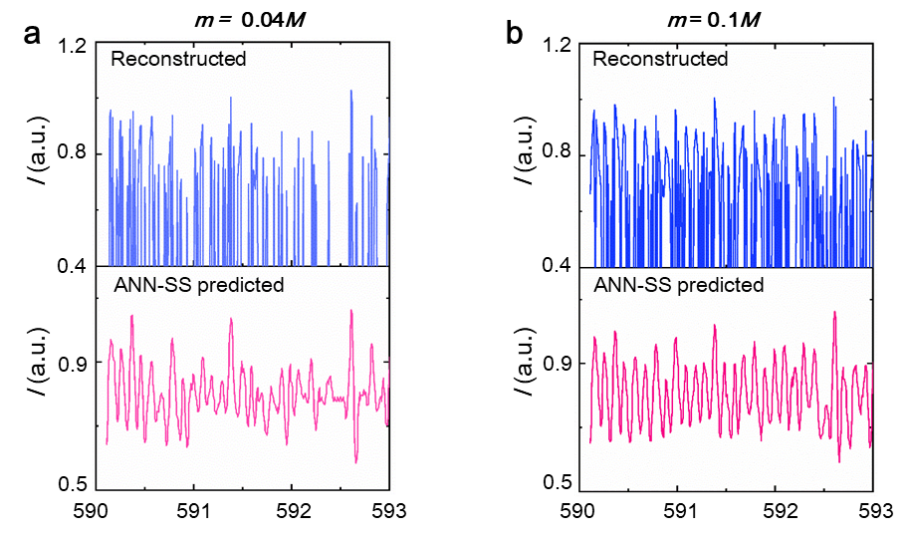


Figure S10 The under sampled spectra reconstructed by traditional method and ANN-SS predicated spectra obtained from different frames.
